# Higher likelihood of 6-months exclusive breastfeeding among HIV infected than uninfected mothers: a household survey in Kenya

**DOI:** 10.1186/s13006-018-0190-9

**Published:** 2018-11-29

**Authors:** John Okanda, George Otieno, John Kinuthia, Pam Kohler, Grace John-Stewart

**Affiliations:** 1 Center for Global Health Research, Kenya Medical Research Institute, Kisumu, Kenya; 2 Kenyatta National Hospital/ University of Nairobi, Nairobi, Kenya; 3 University of Washington, Seattle, WA USA

**Keywords:** Breastfeeding, HIV, Premastication, Kenya

## Abstract

**Background:**

Exclusive breastfeeding (EBF) (breast milk feeding without additional food or drink, except medicine) is associated with deceased risk of postnatal transmission of HIV from mother to child.

**Methods:**

This analysis used data from a household survey in Western Kenya in 2011. Participants were mothers with HIV and uninfected mothers, aged ≥14 years who gave birth in the prior year (ever breastfed) within the Kenya Medical Research Institute/US Centers for Disease Control and Prevention (KEMRI/CDC) Health and Demographic Surveillance System. Data on breastfeeding counseling and knowledge and practices regarding breastfeeding were collected. Rates and correlates of EBF were determined using multivariable logistic regression.

**Results:**

Of 652 mothers enrolled in the study, 435 were included in this analysis. Median age was 28 years among 154 mothers with HIV and 25 years among 281 uninfected mothers. Mothers with HIV were more likely than uninfected mothers to report breastfeeding counseling at a health facility (88.9% vs. 51.6%, respectively, *p* <  0.001) and EBF for 6-months (64.9% versus 34.5%, p <  0.001). Premastication (pre-chewing of food by adults prior to feeding to children) was less prevalent among mothers with HIV (3.9% vs. 13.2% *p* = 0.001) who were also more knowledgeable about potential risk of HIV transmission through premastication (83.1% vs 71.2% *p* = 0.005). Mothers with HIV who EBF for six months were 3.68-fold more likely to report counseling on EBF (aOR 3.68; 95% CI: 1.00,13.70). Uninfected mothers with polygamous marriage, any antenatal care visit, unskilled delivery and delayed breastfeeding initiation (> 1 h) were less likely to practice EBF for six months 62% (aOR 0.38; 95%CI: 0.20,0.94), 72% (aOR 0.28; 95%CI: 0.10,1.00), 54% (aOR 0.46; 95% CI: 0.22,1.00) and 46% (aOR 0.54; 95%CI: 0.30,1.00) respectively.

**Conclusions:**

Mothers with HIV were more likely to report breastfeeding counseling at a health facility, EBF for six months and less likely to practice premastication than uninfected mothers. Lessons learned from breastfeeding counseling in mothers with HIV could be used to improve awareness and change breastfeeding practices for all mothers.

## Background

Optimal nutrition from early stages of life is critical for good physical and mental development and long-term health [1]. As a global public health recommendation, infants should be exclusively breastfed (fed on breast milk without any additional food or drink, not even water, with the exception of medicine) for the first six months of life [2]. After six months, infants should receive safe and nutritionally adequate complementary foods to meet their high nutritional requirements with continued breastfeeding for up to two years or beyond [3]. The World Health Organization (WHO) has recommended that mothers with HIV in settings where replacement feeding is not acceptable and safe, should exclusively breastfeed in the first six months of life followed by safe and appropriate complementary foods [4]. In sub Saharan Africa, mixed breastfeeding (MBF), including administration of foods and liquids in addition to breast milk remains common. Premastication (which is pre-chewing of food by adults prior to feeding to children) is also practiced, [5] further increasing the risk of infant HIV infection, through possible mixing of the virus with the pre-chewed food [6, 7]. Kenya is one of the four HIV ‘high burden’ countries in Africa. The national HIV prevalence among mothers in Kenya aged 15–49 was at 6.5%. Siaya County, where this study was conducted had an overall prevalence of 26.4% among females aged 15–49 years in 2014 [8]. Most Kenyan mothers (99%) breastfeed their children but only 42% breastfeed exclusively for four to five months [9] and breastfeeding continues to contribute to mother-to-child HIV transmission (MTCT) [10]. Mothers can decrease the risk of transmitting HIV to their children if they are on combination antiretroviral treatment (ART) and exclusively breastfeed their infants [4].

The WHO has a global nutrition target to increase the rate of EBF in the first six months up to at least 50% by 2025 [3]. We determined EBF practices and breastfeeding counseling among mothers living in a health and demographic surveillance community in rural western Kenya and compared practices among mothers who have HIV and uninfected mothers.

## Methods

This was a secondary analysis utilizing data from a cross-sectional community level survey on knowledge and uptake of prevention of mother-to-child HIV transmission (PMTCT) services among mothers of child-bearing age conducted between March – June, 2011 in rural Western Kenya. A detailed description of the design and methods has been described elsewhere [11]. In brief, we conducted a community level survey assessing barriers to antenatal care and PMTCT service utilization among mothers with HIV and uninfected mothers, aged ≥14 years (mothers 14–17 are considered emancipated minors) who had delivered in the previous year within the KEMRI/CDC Health and Demographic Surveillance System (KEMRI/CDC HDSS) area in Siaya county, Western Kenya. The HDSS covers 385 villages with a population of approximately 220,000. Three regions situated in Siaya County make up this area: Karemo, Gem and Asembo. All three regions are rural. The HDSS was launched in September 2001 by the US Centers for Disease Control and Prevention (CDC) in collaboration with the Kenya Medical Research Institute (KEMRI) and serves as a community-based platform which provides demographic and health information as well as disease or intervention specific information [12].

Using the HDSS as a sampling frame, we generated a random list of 798 mothers, of whom 652 were eligible, recruited, and enrolled in the main study. Mothers who were HIV positive were purposively oversampled to increase the power to detect associations related to the uptake of PMTCT interventions.

### Data collection and analysis

In this secondary analysis, we assessed infant feeding practices including EBF and premastication feeding. We utilized data from questionnaires regarding demographic information, obstetrical history including breastfeeding, self-reported HIV status and premastication practices. Exclusive breastfeeding duration was assessed by the question; ‘*How long did you exclusively breastfeed?*’ then the interviewer read a script to the mother clarifying the meaning of EBF; ‘*Exclusive breastfeeding means the baby only received breast milk without any additional food or drink, not even water, with the exception of medicine.’*

To assess premastication feeding, the question was framed as; **‘***Have you ever practiced any of the following?’* with multiple choice answers included *‘chewing food for the baby’.* To assess knowledge of possible HIV transmission through premastication, the question was; **‘***Do you think a baby can get HIV through any of the following practices?’* Multiple choice answers included *‘chewing food for the baby’.*

Trained fieldworkers were assigned a list of names and locations for all selected participants and with the help of village reporters (VRs), located the participants, introduced the study, administered informed consent and conducted the interview. Fieldworkers administered the questionnaire via hand-held Personal Digital Assistant (PDAs) using electronic forms (Pendragon Software Corporation, Buffalo Grove, IL). Paper forms were used as a backup.

Data were analyzed using Stata version 11.0 (Corporation, College Station, Texas, USA). Descriptive statistics included frequencies and proportions of mothers who practiced EBF, premastication and those who were knowledgeable about the risk of HIV transmission through premastication. We also reported Pearson’s Chi-square test of association or Fishers exact test for categorical variables and *t* test for continuous variables.

For inferential analysis we performed unadjusted logistic regression stratified by the mother’s HIV status. Variables that had *p* - value of 0.2 or less or set a priori from unadjusted analysis were entered into adjusted regression models to assess significant predictors. Odds Ratio, *p* - values and 95% confidence intervals were reported.

## Results

The general characteristics of 652 mothers enrolled in the main study have been previously reported [13]. This analysis was limited to 435 mothers who reported to have ever breastfed their infants and provided information on their HIV status and duration of breastfeeding. The median age of mothers with HIV was 28 years (IQR 24–32), older than that of uninfected mothers (median 25 years [IQR 22–30], *p* <  0.001) (Table 1). Most mothers had less than secondary education (87.7% of HIV infected and 86.8% of HIV uninfected mothers). Over half of the mothers (51.9% HIV infected and 59.8% HIV uninfected) reported home deliveries.

**Table 1:** Sociodemographic and breastfeeding characteristics of mothers with HIV and without HIV

Variables	HIV uninfected (*n* = 281)	HIV infected (*n* = 154)	
	*n* (%) or median (IQR)	*n* (%) or median (IQR)	*p* -value
Age	25 (22–30)	28 (24–32)	< 0.001
Marital status			
Married (monogamous)	226 (80.4)	112 (72.7)	
Married (polygamous)	38 (13.5)	24 (15.6)	
Single	15 (5.3)	5 (3.4)	
Widowed	2 (0.7)	13 (8.4)	< 0.001
Employed	129 (46.1)	53 (34.6)	0.025
Primary education or less	243 (86.8)	135 (87.7)	0.881
Any antenatal care visit	268 (95.4)	148 (96.1)	0.810
Home delivery^a^	168 (59.8)	80 (51.9)	0.129
Delivery care			
No assistance	59 (21.0)	31 (20.4)	
Skilled assistance	117 (41.6)	75 (49.3)	
Unskilled assistance	105 (37.4)	46 (30.3)	0.256
Received breastfeeding counseling at clinic	145 (51.6)	136 (88.9)	< 0.001
Initiation of breastfeeding			
< 1 h after delivery	188 (66.9)	98 (63.6)	
1 h – 1 day after delivery	83 (29.5)	52 (33.8)	
> 1 day after delivery	10 (3.6)	4 (2.6)	0.651
Exclusive breastfeeding duration			
< 6 mo	184 (65.5)	54 (35.1)	
6 mo	97 (34.5)	100 (64.9)	< 0.001
Median duration of exclusive breastfeeding	4 (3–6)	6 (4–6)	< 0.001
Ever premasticated	37 (13.2)	6 (3.9)	0.001
Knowledge of HIV transmission through premastication	200 (71.2)	128 (83.1)	0.005

^a^Home delivery is not included in multivariable analysis because it is collinear with delivery care

Mothers with HIV were significantly more likely than uninfected mothers to report receiving breastfeeding counseling at a health facility (88.9% vs. 51.6%, *p* <  0.001). Over 60% of mothers reported breastfeeding in the first hour after birth with similar rates in both HIV and uninfected mothers (*p* = 0.651). Mothers with HIV were also more likely than uninfected mothers to report EBF for six months (64.9% versus 34.5%, *p* <  0.001) (Table 1).

Premastication feeding was reported by fewer mothers with HIV than uninfected mothers (3.9% vs. 13.2%, *p* = 0.001). Mothers with HIV were more knowledgeable regarding premastication feeding as a possible route for HIV transmission (83.1% vs. 71.2%, *p* = 0.005) (Table 1).

Table 2 summarizes regression analysis of factors associated with EBF for six months among mothers with HIV and uninfected mothers. In adjusted analysis, uninfected mothers who were in polygamous marriages were 62% less likely to practice EBF for six months aOR 0.38 (95% CI 0.20, 0.94). Uninfected mothers who made at least one antenatal care clinic visit were 72% less likely to practice EBF for six months, aOR 0.28 (95% CI 0.10, 1.00). Similarly, uninfected mothers who were assisted to deliver by unskilled persons were 54% less likely to practice EBF for six months, aOR 0.46 (95% CI 0.22, 1.00). We also found that uninfected mothers who initiated breastfeeding after one hour of birth were 46% less likely to practice EBF for six months, aOR 0.54 (95% CI 0.30, 1.00).

**Table 2:** Factors associated with exclusive breastfeeding for 6 months among mothers without HIV and with HIV

	HIV uninfected mothers (*n* = 279)^a^				HIV infected mothers (*n* = 147)^a^			
	Unadjusted Odds Ratio		Adjusted Odds Ratio		Unadjusted Odds Ratio		Adjusted Odds Ratio	
	[OR](95% CI)	*P*-value	[OR](95% CI)	*P*-value	[OR](95% CI)	*P*- value	[OR] (95% CI)	*P*-value
Age	0.97 (0.93, 1.0)	0.097	0.96 (0.91, 1.0)	0.081	1.04 (1.00, 1.12)	0.128	1.04 (1.00, 1.12)	0.345
Marital status								
Married (monogamous)	1				1		1	
Married (polygamous)	0.40 (0.17, 1.00)	0.040	0.38 (0.20, 0.94)	0.036	1.07 (0.42, 2.72)	0.889	0.82 (0.30, 2.31)	0.701
Single	2.04 (0.72, 5.90)	0.182	2.14 (0.63, 7.21)	0.222	0.36 (0.06, 2.22)	0.269	0.23 (0.02, 2.60)	0.234
Widowed	1.79 (0.11, 29.0)	0.682	3.02 (0.20, 53.00)	0.463	1.20 (0.40, 4.20)	0.771	1.24 (0.32, 4.83)	0.759
Any antenatal care visit	0.43 (0.14, 1.33)	0.143	0.28 (0.10, 1.00)	0.048	1.90 (0.40, 9.80)	0.441	1.91 (0.30, 12.80)	0.505
Delivery care								
No assistance	1				1			
Skilled assistance	1.17 (0.61, 2.23)	0.633	0.97 (0.50, 2.00)	0.942	0.74 (0.30, 1.90)	0.528	0.89 (0.30, 2.63)	0.834
Unskilled assistance	0.58 (0.30, 1.20)	0.122	0.46 (0.22, 1.00)	0.042	0.41 (0.20, 1.12)	0.081	0.55 (0.20, 1.72)	0.306
Received breastfeeding counseling at clinic	1.28 (0.80, 2.10)	0.322	1.41 (0.83, 2.41)	0.202	3.96 (1.40, 11.43)	0.011	3.68 (1.00, 13.70)	0.052
Initiation of breastfeeding								
< 1 h after delivery	1							
1 h - 1 day after delivery	0.67 (0.40, 1.17)	0.161	0.54 (0.30, 1.00)	0.049	0.84 (0.42, 1.70)	0.633	0.97 (0.50, 2.60)	0.952
> 1 day after delivery	0.41 (0.10, 2.00)	0.270	0.32 (0.06, 1.84)	0.204	0.16 (0.02, 1.70)	0.121	0.24 (0.02, 2.90)	0.261
Knowledge of HIV transmission through premastication	1.16 (0.70, 2.02)	0.587	1.04 (0.60, 1.90)	0.896	1.75 (0.80, 4.13)	0.197	2.17 (0.81, 5.83)	0.123

^a^Among 279 HIV uninfected and 147 HIV infected mothers with available data at time point of interest

Among mothers with HIV, there was some evidence that receiving breastfeeding counseling at health facility was associated with EBF for six months, aOR 3.68 (95% CI 1.00, 13.70) (Table 2).

## Discussion

Most mothers in this study breastfed in early postpartum period and initiated breastfeeding within an hour of delivery. Mothers with HIV were more likely than uninfected mothers to report breastfeeding counseling at health facilities and to practice EBF for six months. Mothers with HIV who practiced EBF for six months were more likely to have received breastfeeding counseling at health facility. This study identified several gaps in counselling and support for infant feeding practices that affected adherence to EBF especially among HIV uninfected mothers. These included being in polygamous marriages, fewer antenatal care clinic visits, unskilled delivery assistance and initiation of breastfeeding after one hour of birth. Together, this data suggests that health facility counseling to encourage EBF was effective in changing breastfeeding practices of mothers with HIV.

Over 88% of mothers with HIV received breastfeeding counseling at the health facility and mothers who receiving counseling had 3.68-fold increased odds of EBF for at least six months. Only half of uninfected mothers reported that they received breastfeeding counseling at the health facility. The findings suggest that breastfeeding counseling influences EBF duration and that health facilities may preferentially emphasize breastfeeding counseling for mothers with HIV. While it is reassuring that mothers with HIV receive breastfeeding counseling and appear to have better breastfeeding practices as a result, the finding suggests that there is room to improve breastfeeding counseling for all mothers. Increased counseling to emphasize sustained EBF are necessary for all mothers in maternal child health programs.

Several recent studies have suggested that breastfeeding counseling during the perinatal period is effective in promoting exclusive breastfeeding [14–17]. Breastfeeding counseling during pregnancy, immediately after childbirth and at key moments in the postnatal period have been associated significantly with EBF practices [2, 16, 17]. Using peer counselors is one effective way to offer breastfeeding counseling [18] and has been associated with less prelacteal feeding [19]. A recent clinic-level study to investigate the impact of a counseling intervention on EBF among mothers with HIV in Nairobi found no significant difference in EBF prevalence between the cases and controls, however, the EBF prevalence was found to be high in the non-intervention arm of this study, making it difficult to evaluate the impact of counseling [20]. A systematic review of breastfeeding promotion intervention and breastfeeding practices, also suggests that breastfeeding counseling and/or support increased EBF rates [14].

We found similar rates (60%) of breastfeeding initiation soon after birth in other studies in Tanzania 65%, Kenya 76% and Uganda 55% [16, 21, 22]. The Kenya Demographic and Health Survey (DHS) of 2014 reported that 62% of mothers had early initiation of breastfeeding with mothers in the lowest wealth quintile more likely to initiate early breastfeeding than those in the higher wealth quintile [9]. In contrast, a multi-site study in Burkina Faso, Uganda and South Africa by the PROMISE-EBF group and another in Northwest Ethiopia, observed that majority of mothers did not initiate breastfeeding during the first hour after birth [19, 23].

The prevalence of six month EBF, particularly among mothers with HIV, is higher than in other studies from this region. This may reflect better uptake of PMTCT services among the mothers with HIV in the study area. A community-based survey in Northeastern Tanzania reported prevalence of six months EBF of 24.1%, lower than in both HIV infected and uninfected mothers in our study [16]. Advanced maternal age and knowledge of exclusive breastfeeding advantages were associated with six months EBF in this study [16]. A study in Northwest Ethiopia reported six month EBF prevalence of 31% [23]. The Kenya 2014 DHS reported EBF prevalence between four to five months of age of 42%, which was higher than among mothers with HIV, but lower than rate in our cohort [9]. Our findings of higher EBF prevalence in HIV-infected than uninfected mothers is consistent with a prior study in the same region [21].

Few mothers with HIV practiced premastication and a high proportion of them reported knowledge that premastication was a potential route for HIV transmission. Knowledge regarding HIV risk of premastication was higher in HIV infected than uninfected mothers, while practice of premastication was lower in mothers with HIV than uninfected mothers. It is encouraging that mothers with HIV avoided premastication, however, it is not clear how mothers became aware of the risk, since premastication education or counseling was not routinely practiced to the best of our knowledge.

This study had several strengths. It was conducted in the community, enabling us to reach mothers who would not have attended a health facility. Working within the HDSS that has information on HIV status of residents enabled us to oversample mothers with HIV for this study.

We had limitations that included the use of self-reported information on feeding practices, HIV status and health facility attendance that could introduce biases. We also acknowledge that the findings are from over five years ago and some aspects of MCH and PMTCT systems and breastfeeding counseling may have changed during this time.

## Conclusion

We found better exclusive breastfeeding practices in mothers with HIV than uninfected mothers in this community-based survey. The study suggests that breastfeeding counseling for mothers with HIV had a positive impact on EBF practices. It is important to improve EBF practices among all mothers and this may require refresher training and improved breastfeeding counseling for all mothers.

## Abbreviations

CDC: US centres for disease control and prevention
EBF: Exclusive breastfeeding
HDSS: Health and demographic surveillance system
HIV: Human immunodeficiency virus
KEMRI: Kenya medical research institute
MBF: Mixed breastfeeding
PMTCT: Prevention of mother-to-child transmission

## References

[1] World Health Organization (2014). Comprehensive Implementation Plan on Maternal, Infant and Young Child Nutrition In.

[2] World Health Organization. Essential Nutrition Actions: Improving Maternal, Newborn, Infant and Young Child Health Nutr In: Geneva, WHO; 2013. 25473713

[3] World Health Organization (2014). Global Nutrition Targets 2025: Breastfeeding Policy Brief (WHO/NMH/NHD/14.7).

[4] World Health Organization (2016). Guideline: updates on HIV and infant feeding: the duration of breastfeeding, and support from health services to improve feeding practices among mothers living with HIV.

[5] Pelto GH, Zhang Y, Habicht JP (2010). Premastication: the second arm of infant and young child feeding for health and survival?. Maternal and Child Nutrition.

[6] Gaur AH, Dominguez KL, Kalish ML, Rivera-Hernandez D, Donohoe M, Brooks JT (2009). Practice of feeding premasticated food to infants: a potential risk factor for HIV transmission. Pediatrics.

[7] Labrana Y, Alvarez AM, Villarroel J, Wu E (2013). Premastication: a new way of transmitting HIV. First pediatric case reported in Chile. Revista Chilena de Infectologia.

[8] National AIDS and STI Control Programme MoH: Kenya HIV Estimates 2015. In*.* Nairobi, Kenya: NASCOP; 2016.

[9] Government of Kenya: Kenya demographic and health survey (DHS) 2014. In*.* Nairobi, Kenya: Kenya National Bureau of Statistics; 2014.

[10] Avert: Children, HIV and AIDS: Fact sheet. 2017. Available at https://www.avert.org/professionals/hiv-social-issues/key-affected-populations/children

[11] Kohler PK, Okanda J, Kinuthia J, Mills LA, Olilo G, Odhiambo F (2014). Community-based evaluation of PMTCT uptake in Nyanza Province, Kenya. PLoS One.

[12] Odhiambo FO, Laserson KF, Sewe M, Hamel MJ, Feikin DR, Adazu K (2012). Profile: the KEMRI/CDC health and demographic surveillance system--Western Kenya. Int J Epidemiol.

[13] Kohler PK, Okanda J, Kinuthia J, Mills LA, Olilo G, Odhiambo F (2014). Community-based evaluation of PMTCT uptake in Nyanza province, Kenya. PLoS One.

[14] Haroon S, Das JK, Salam RA, Imdad A, Bhutta ZA (2013). Breastfeeding promotion interventions and breastfeeding practices: a systematic review. BMC Public Health.

[15] Liben MLGY, Adugnew M, Asrade A, Adamie B, Gebremedin E, Melak Y (2016). Factors associated with exclusive breastfeeding practices among mothers in dubti town, afar regional state, Northeast Ethiopia: a community based cross-sectional study. Int Breastfeed J.

[16] Maonga AR, Mahande MJ, Damian DJ, Msuya SE (2016). Factors affecting exclusive breastfeeding among women in Muheza district Tanga northeastern Tanzania: a mixed method community based study. Matern Child Health J.

[17] Okanda JO, Borkowf CB, Girde S, Thomas TK, Lecher SL (2014). Exclusive breastfeeding among women taking HAART for PMTCT of HIV-1 in the Kisumu breastfeeding study. BMC Pediatr.

[18] Chola L, Fadnes LT, Engebretsen IM, Nkonki L, Nankabirwa V, Sommerfelt H (2015). Cost-effectiveness of peer counselling for the promotion of exclusive breastfeeding in Uganda. PLoS One.

[19] Engebretsen IM, Nankabirwa V, Doherty T, Diallo AH, Nankunda J, Fadnes LT (2014). Early infant feeding practices in three African countries: the PROMISE-EBF trial promoting exclusive breastfeeding by peer counsellors. Int Breastfeed J.

[20] Bosire RBB, Aluisio A, Hughes JP, Nduati R, Kiarie J, Chohan BH (2016). High rates of exclusive breastfeeding in both arms of a peer counseling study promoting EBF among HIV-infected Kenyan women. Breastfeed Med.

[21] Gewa CA, Oguttu M, Savaglio L (2011). Determinants of early child-feeding practices among HIV-infected and noninfected mothers in rural Kenya. J Hum Lact.

[22] Somé EN, Engebretsen IMS, Nagot N, Meda N, Lombard C, Vallo R (2017). Breastfeeding patterns and its determinants among mothers living with human Immuno-deficiency virus −1 in four African countries participating in the ANRS 12174 trial. Int Breastfeed J.

[23] Biks GA, Tariku A, Tessema GA (2015). Effects of antenatal care and institutional delivery on exclusive breastfeeding practice in Northwest Ethiopia: a nested case-control study. Int Breastfeed J.

